# Changes in the Hemagglutinin and Internal Gene Segments Were Needed for Human Seasonal H3 Influenza A Virus to Efficiently Infect and Replicate in Swine

**DOI:** 10.3390/pathogens11090967

**Published:** 2022-08-25

**Authors:** Daniela S. Rajao, Eugenio J. Abente, Joshua D. Powell, Marcus J. Bolton, Phillip C. Gauger, Bailey Arruda, Tavis K. Anderson, Troy C. Sutton, Daniel R. Perez, Amy L. Vincent Baker

**Affiliations:** 1 Department of Population Health, College of Veterinary Medicine, University of Georgia, Athens, GA 30602, USA; 2 Virus and Prion Research Unit, National Animal Disease Center, USDA-ARS, Ames, IA 50010, USA; 3 Department of Veterinary Diagnostic & Production Animal Medicine, Iowa State University, Ames, IA 50011, USA; 4 Department of Veterinary and Biomedical Sciences, The Pennsylvania State University, University Park, PA 16802, USA

**Keywords:** influenza, swine, human, surface genes, internal genes, adaptation

## Abstract

The current diversity of influenza A viruses (IAV) circulating in swine is largely a consequence of human-to-swine transmission events and consequent evolution in pigs. However, little is known about the requirements for human IAVs to transmit to and subsequently adapt in pigs. Novel human-like H3 viruses were detected in swine herds in the U.S. in 2012 and have continued to circulate and evolve in swine. We evaluated the contributions of gene segments on the ability of these viruses to infect pigs by using a series of in vitro models. For this purpose, reassortant viruses were generated by reverse genetics (rg) swapping the surface genes (hemagglutinin-HA and neuraminidase-NA) and internal gene segment backbones between a human-like H3N1 isolated from swine and a seasonal human H3N2 virus with common HA ancestry. Virus growth kinetics in porcine intestinal epithelial cells (SD-PJEC) and in ex-vivo porcine trachea explants were significantly reduced by replacing the swine-adapted HA with the human seasonal HA. Unlike the human HA, the swine-adapted HA demonstrated more abundant attachment to epithelial cells throughout the swine respiratory tract by virus histochemistry and increased entry into SD-PJEC swine cells. The human seasonal internal gene segments improved replication of the swine-adapted HA at 33 °C, but decreased replication at 40 °C. Although the HA was crucial for the infectivity in pigs and swine tissues, these results suggest that the adaptation of human seasonal H3 viruses to swine is multigenic and that the swine-adapted HA alone was not sufficient to confer the full phenotype of the wild-type swine-adapted virus.

## 1. Introduction

Influenza is caused by a zoonotic pathogen that infects many species of birds and mammals, posing a threat to both human and animal health [1]. Influenza A viruses (IAV) are enveloped, single-stranded segmented RNA viruses that belong to the *Orthomyxoviridae* family. The surface glycoproteins, hemagglutinin (HA) and neuraminidase (NA), are the main targets for host immunity and define the subtypes to IAVs [2]. Although only H1N1, H1N2, and H3N2 IAV subtypes are endemic in pigs [3], numerous HA and NA lineages co-circulate among swine herds globally. As an example, at least 15 distinct H1 and H3 phylogenetic clades co-circulate in North American swine populations currently [3,4]. This phylogenetic diversity is in part a result of a dramatic evolution of swine IAVs following the introduction of a triple reassortant virus containing avian-, swine-, and human-origin internal gene segments in the late 1990s [3,5].

Overall, the bidirectional transmission of IAVs between humans and swine has played a major role in these evolutionary events and greatly contributed to the majority of the genetically and antigenically distinct viruses circulating in pigs [6,7]. All the HA phylogenetic clades currently circulating in pigs in the U.S. have evolved from a human IAV. One of these human-to-swine spillover events was detected in the last decade and led to the establishment of the new 2010.1 H3 lineage in U.S. pigs [8]. Although human-to-swine transmission events are relatively common [6,7,9], wholly human seasonal viruses do not always become established in pigs; rather, individual viral gene segments become incorporated into endemic swine strains through reassortment [8,10,11]. Interestingly, in many of these human-to-swine spillover events, the human-origin gene segments that are maintained in the swine population, particularly the surface genes, show substantial genetic differences from the seeding human strains [8,9], suggesting that changes are required for the persistence of such viruses in the swine host.

The HA has a pivotal role in virus binding to the host cell and has been pointed as one of the most impactful factors affecting host specificity [12,13]. The HA binds to glycan receptors on the surface of host epithelial cells with different receptor specificity: human viruses preferably bind to sialic acid (SA) residues linked to the penultimate galactose by α2,6 linkages and avian influenza viruses preferentially recognize α2,3-linked SA [14,15,16]. Amino acid substitutions in or near the receptor binding site in the HA can change the receptor preference from α2,3 to α2,6-SA linkages [16,17]. Pigs possess both receptors in their respiratory tract in a similar distribution as the human respiratory tract [18,19]. Hence, swine and human adapted IAV tend to have similar glycan-binding preference to α2,6-linked SA [20], but other structural features of glycans (e.g., type, length, etc.) may affect receptor-specificity and impose barriers during the adaptation between the two species [21]. Susceptibility of the virus to different innate immune components such as collectins, pattern recognition receptors (PRR), and interferon-stimulated genes (ISGs) have also been shown to restrict host range, since these factors may show different functionalities in different host species [21,22,23,24]. Additionally, changes in the PB2 gene were shown to affect the optimal temperature of replication of different IAVs and, therefore, affect their replication in different physiological host conditions [25,26,27]. Although these and other mechanisms have been shown to contribute to host specificity of IAVs, the molecular basis for IAV host-range restriction is not fully understood, particularly for human IAV in pigs.

Understanding these mechanisms of IAV adaptation to the swine species will allow for more rapid identification of human viruses that have the potential to become established in pigs. Further, understanding the features in IAVs that impact human-to-swine (reverse zoonosis) and swine-to-human (zoonosis) transmission is of public health and pandemic preparedness importance. Here, we investigated the viral and host factors that could be associated with the adaptation of human seasonal IAV to the swine host. Our results indicate that the swine-adapted HA increased binding and entry of the virus to swine cells, which consequently improved replication in this system. However, swine-origin internal genes also played a role in IAV replication in swine cells. Thus, adaptation of human H3N2 to swine required mutation and reassortment involving multiple gene segments.

## 2. Results

### 2.1. Replication Efficiency of Reassortant Viruses in Swine Cells Depended on the HA or Internal Gene Constellation

**Figure 1:**
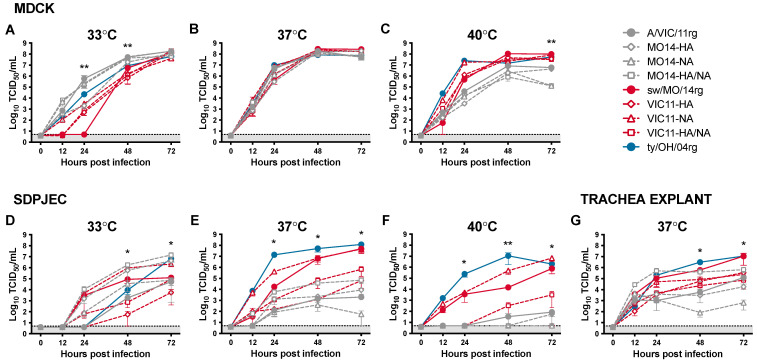
In vitro replication kinetics of reassortant viruses at different temperatures. Viral growth kinetics of reassortant viruses with swapped HA/NA segments between A/swine/Missouri/A01410819/2014 H3N1 (sw/MO/14) and A/Victoria/361/2011 H3N2 (A/VIC/11) in (**A**–**C**) MDCK, (**D**–**F**) SD-PJEC at 33 °C, 37 °C, and 40 °C, and (**G**) trachea explants at 37 °C. Cells were infected at MOI = 0.01 and supernatants were collected at 0, 12, 24, 48, and 72 h post-infection (hpi). Viral titers were quantified by TCID_50_/mL. Plotted data are shown as mean TCID_50_/mL titers ± standard error of the mean of two independent experiments, each conducted in triplicates. * *p* < 0.05, ** *p* < 0.01. Colors correspond to the internal gene cassettes of grey—A/VIC/11, red—sw/MO/14, and blue—ty-OH/04 serving as a positive control. The shaded gray area indicates values below the limit of detection of the assay.

The role of the surface glycoproteins and internal gene cassettes on viral replication was investigated by inoculating Madin-Darby canine kidney (MDCK) cells and South Dakota porcine jejunum epithelial cells (SD-PJEC) at low MOI and incubating at 33 °C, 37 °C, and 40 °C to emulate biologic temperatures of human versus swine upper and lower respiratory tracts (Figure 1). Six viruses (Table 1) were generated by reverse genetics (rg) with swapped genes from A/swine/Missouri/A01410819/2014 and A/Victoria/361/2011: MO14-HA, MO14-NA, MO14-HA/NA, VIC11-HA, VIC11-NA, VIC11-HA/NA, in addition to parental viruses sw/MO/14rg and A/VIC/11rg, and ty/OH/04 control virus. All reassortant viruses showed similar replication kinetics in MDCK cells at 37 °C (Figure 1B). However, when using the SD-PJEC swine cell line at this temperature, they showed different replication profiles: by switching the sw/MO/14 HA gene with the human seasonal A/VIC/11 HA gene, there was a significant loss in replication efficiency compared to the viruses containing the sw/MO/14 HA (Figure 1E). However, the HA gene alone was not enough for the viruses with the sw/MO/14 HA gene and A/VIC/11 internal genes (MO14-HA and MO14-HA/NA, Figure 1E open gray diamond and open gray square, respectively) to reach the full replication efficiency and viral titers of the sw/MO/14rg whole virus (Figure 1E, solid red circle), and they showed only mildly increased replication compared to the A/VIC/11 whole virus (Figure 1E, solid gray circle). Interestingly, the virus with the sw/MO/14 HA gene and A/VIC/11 N2 gene (Figure 1E, open red triangle) showed replication similar to that of the sw/MO/14rg whole virus, suggesting that the N1 gene did not have a major role in the replication efficiency of the human-origin swine viruses.

At 33 °C, viruses with the A/VIC/11 internal genes (gray) replicated more efficiently overall than viruses with the sw/MO/14 internal genes (red; Figure 1A,D), although viruses with the A/VIC/11 HA gene (A/VIC/11rg and MO14-NA, solid gray circle and open gray triangle, respectively) showed lower replication efficiency in the swine cell line compared to viruses with sw/MO/14 HA (MO14-HA and MO14-HA/NA, open gray diamond and open gray square, respectively). The opposite was observed at 40 °C, as viruses with A/VIC/11 internal genes (gray) showed decreased replication compared to viruses with the sw/MO/14 internal genes (red), and the viruses with the A/VIC/11 HA gene replicated poorly in the swine cells like what was observed at 33 °C (Figure 1C,F). Most viruses with the sw/MO/14 internal genes (red) generally showed higher replication efficiency in swine ex-vivo trachea explants compared to the viruses with A/VIC/11 internal genes (gray), except for MO14-HA/NA (open gray square), which replicated similarly to the whole virus sw/MO/14rg (solid red circle, Figure 1G).

### 2.2. Reassortant Viruses with Swine-Adapted HA Show Abundant Binding to Swine Respiratory Tissue

**Figure 2:**
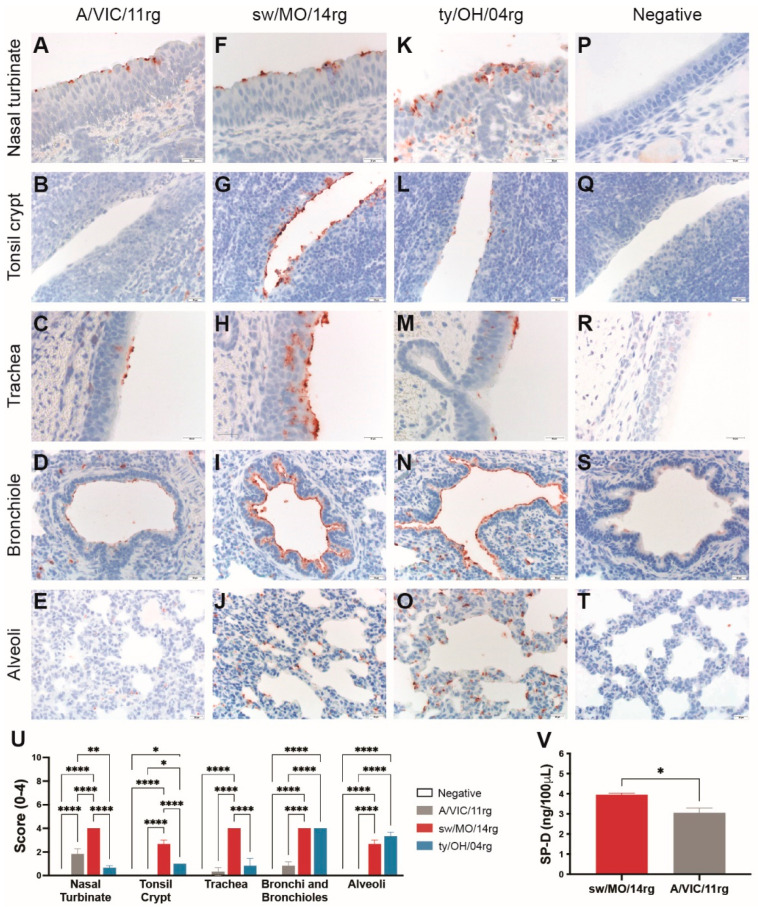
Attachment profile and susceptibility to surfactant protein D (SP-D) of reassortant viruses. Virus attachment detected by virus histochemistry staining of (**A**–**E**) A/Victoria/361/2011 (A/VIC/11rg), (**F**–**J**) A/swine/Missouri/A01410819/2014 (sw/MO/14rg), and (**K**–**O**) A/turkey/Ohio/313053/ 2004 (ty/OH/04rg) to formalin-fixed, paraffin-embedded swine respiratory tissue, 200–400 X. Negative controls were included for each tissue (**P**–**T**). Nasal turbinate, tonsil crypt, trachea, and lung from three IAV-naïve pig were incubated with FITC-labeled viruses and then stained with peroxidase-labeled biotin system with 3-Amino-9-ethylcarbazole (AEC) substrate and hematoxylin counterstain. (**U**) Scores for virus binding to the respiratory tract detected by virus histochemistry staining. Values are shown as average scores ± standard error of the mean, ranging from 0–4. * *p* < 0.05, ** *p* < 0.01, **** *p* < 0.0001. (**V**) Levels of porcine surfactant protein D (pSP-D) hemagglutination inhibition of turkey red blood cells. Inhibition values indicate the lowest concentration of pSP-D capable of inhibiting hemagglutination of 4 HAU of sw/MO/14rg or A/VIC/11rg in equal volumes. Plotted data are shown as mean pSP-D concentrations ± standard error of the mean of two independent experiments, each conducted in triplicate. * *p* < 0.05.

We investigated the binding profile of swine adapted surface genes by virus histochemistry using swine respiratory tissue. The human-origin H3N1 virus containing swine-adapted surface genes (sw/MO/14) bound abundantly to epithelial cells of the nasal turbinates (Figure 2F), tonsil crypts (Figure 2G), trachea (Figure 2H), bronchioles (Figure 2I), and alveoli (Figure 2J) of pigs (quantitated in Figure 2U). The control ty/OH/04 also bound throughout the swine respiratory tract, although more abundantly to the lower respiratory tract epithelial cells (Figure 2K–O). However, the human seasonal virus (A/VIC/11) bound to much fewer cells, showing moderate binding to the nasal turbinate epithelial cells (Figure 2A,U) and almost no binding to the lower respiratory tract (Figure 2D,E,U).

### 2.3. The Human Seasonal Virus HA Was More Susceptible to Porcine Surfactant Protein-D (pSP-D) Than the Swine-Adapted Virus HA

To investigate the susceptibility of the human- or swine-adapted IAVs to pSP-D, we performed an HAI assay using recombinant pSP-D. The virus containing the human seasonal HA (A/VIC/11rg) showed greater susceptibility to pSP-D, as virus was neutralized at significantly lower pSP-D concentrations compared to the virus containing swine-adapted HA (sw/MO/14rg; Figure 2V).

### 2.4. Attachment and Entry into Swine Cells Were Greater in Reassortant Viruses with Swine-Adapted HA

**Figure 3:**
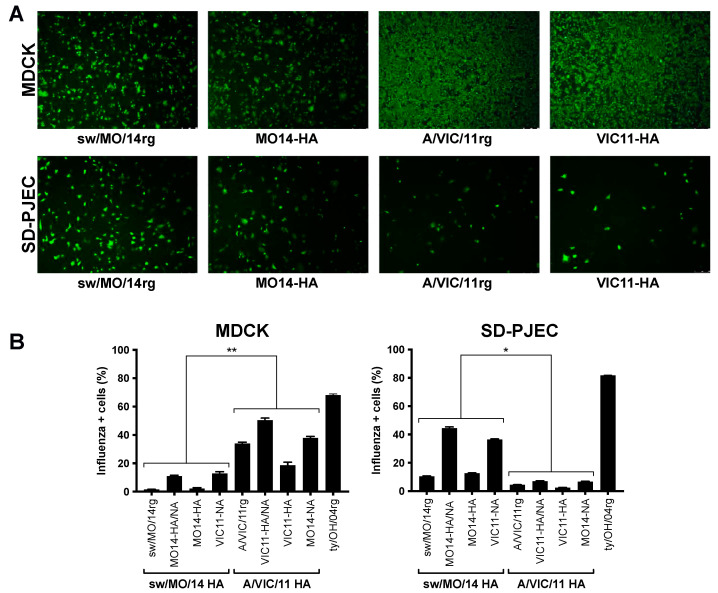
Entry of reassortant viruses in MDCK and SD-PJEC cells. Entry efficiency of viruses containing A/swine/Missouri/A01410819/2014 HA (sw/MO/14 and MO14-HA) and A/Victoria/361/2011 HA (A/VIC/11 and VIC11-HA) detected by immunofluorescence at 1 h post infection (hpi), 5X (**A**) or measured by FACS on a flow cytometer at 1 hpi (**B**) in MDCK and SD-PJEC cells. Plotted data are shown as average number of influenza-positive cells ± standard error of the mean of two independent experiments, each conducted in triplicates. Viruses with the same HA were grouped for statistical comparison. * *p* < 0.05, ** *p* < 0.01.

To further explore if the swine-adapted surface glycoproteins or internal genes cassette played a role in virus attachment and entry, we compared MDCK versus SD-PJEC cells at 1 h post-infection via immunofluorescence or flow cytometry. Viruses with the swine-adapted (sw/MO/14rg and MO14-HA) and human seasonal (A/VIC/11rg and VIC11-HA) HA genes showed similar attachment and entry in MDCK cells. However, in SD-PJEC, sparser signals were detected for viruses with the human seasonal HA compared to the viruses with the swine adapted HA (Figure 3A). Consistently more MDCK cells were influenza-positive for viruses containing A/VIC/11 HA compared to those containing sw/MO/14 HA (Figure 3B) while incubation with viruses containing swine-adapted HA gene resulted in higher numbers of influenza-positive SD-PJEC compared to viruses with a human adapted HA gene (Figure 3B).

### 2.5. The Polymerase Complex of the Human Seasonal Virus Showed Increased Activity at All Temperatures

**Figure 4:**
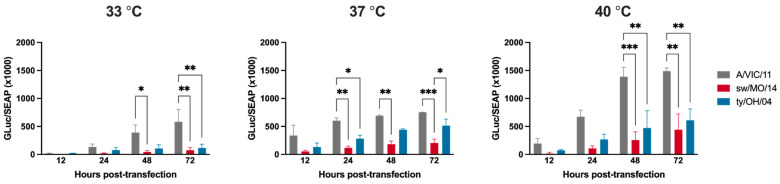
Virus polymerase activity in human-origin and swine-origin polymerase complexes. MDCK-SIAT1 cells were transfected with plasmids encoding the A/Victoria/361/2011 (A/VIC/11rg, grey), A/swine/Missouri/A01410819/2014 (sw/MO/14rg, red), or A/turkey/Ohio/313053/ 2004 (ty/OH/04rg, blue) polymerase complex (PB1, PB2, PA, NP) and the reporter plasmid encoding Gaussia luciferase in the pHW-SP vector. Transfection efficiency was normalized using a plasmid encoding the secreted alkaline phosphatase (SEAP), analyzed at 12 h post transfection. Cell supernatant was collected at 12, 24, 48, and 72 h post-transfection. Plotted data are shown as mean ± standard error of the mean of two independent experiments, each conducted in triplicates. * *p* < 0.05, ** *p* < 0.01, *** *p* < 0.001.

To explore if the swine-adapted internal gene backbone played a role in virus replication at different temperatures, we compared the polymerase activity of the different viral ribonucleoprotein (vRNP) complexes composed of PB2, PB1, PA, and NP segments in MDCK-SIAT1 cells using a minigenome reconstitution assay. The A/VIC/11 polymerase complex plasmids demonstrated significantly higher levels of Gluc reporter replicon activity at 48 and 72 h post transfection at all temperatures tested compared to the swine-adapted sw/MO/14 and ty/OH/04 polymerase complexes (Figure 4). In contrast to what was observed in the replication kinetics analysis, in which viruses with A/VIC/11 backbone showed lower replication at 40 °C, the levels of Gluc reporter activity for the A/VIC/11 polymerase complex plasmids were highest at this temperature (Figure 4).

## 3. Discussion

The diversity of swine IAVs that currently co-circulate in pigs in the United States is a consequence of human-to-swine transmission events [9]. Although human-origin viruses and/or viral segments only sporadically become endemic in pigs, the lineages that have become established, such as the triple reassortant internal gene (TRIG) constellation, have greatly contributed to virus evolution and the current problems producers are faced with regarding effective vaccination and control strategies [3,9]. Despite the importance of reverse zoonotic episodes, little is known about the circumstances under which human IAVs transmit and become adapted to swine. We previously reported a spillover of human seasonal IAV that became endemic in U.S. swine herds in the past decade, leading to establishment of the 2010.1 lineage [8,28]. The earliest H3N1 viruses isolated from pigs acquired a swine-adapted internal gene constellation in addition to numerous amino acid changes in the HA gene segment compared to the nearest human seasonal virus ancestor [8]. Here, we found that the adaptation of these novel human H3 viruses to pigs was a multigenic trait and that the swine-adapted HA alone was not sufficient to confer the full phenotype of the wild-type parental virus. Rather, a sequence of evolutionary events may have allowed for these viruses to adapt and persist in swine.

We previously showed that following human-to-swine spillover of the 2010.1 lineage, different generations of this HA clade of human-origin viruses replicated and transmitted efficiently in pigs, although infection with the first detected virus resulted in an intermediate level of pathology and replication [8,28]. Using a set of reassortant viruses with swapped surface gene segments (HA and/or NA) in the internal gene backgrounds of a swine-adapted virus (sw/MO/14) or a human seasonal virus (A/VIC/11) [8], we previously showed that viruses with the HA of sw/MO/14 replicated more efficiently in the lower respiratory tract of infected pigs than viruses with the A/VIC/11 HA. However, viruses containing the human seasonal HA paired with the internal gene segments of sw/MO/14 replicated to some extent in the upper respiratory tract. Nevertheless, transmission was only observed for the wild-type swine-adapted virus, suggesting that a combination of viral gene segments is needed for replication and transmission in pigs [8]. Here, we tested reassortant viruses with swapped HA and/or NA in a series of in vitro assays to better understand the role of surface and internal gene segments on the adaptation of these human-origin H3 viruses to pigs. Switching the sw/MO/14 HA with the A/VIC/11 HA (VIC11-HA, VIC11-HA/NA, MO14-NA) led to significant loss in replication efficiency in a swine cell line or swine trachea explants. The viruses with the swine-adapted sw/MO/14 HA and A/VIC/11 internal gene segments (MO14-HA and MO14-HA/NA) showed slight increase in replication compared to the A/VIC/11 whole virus but did not reach the replication efficiency of the wholly sw/MO/14 virus. These data confirm our previous findings and support the proposition that the internal genes also play a crucial role for a human-origin IAV to reach its full replication efficiency in pigs; however, this may not be due to increased polymerase activity at the basal body temperature of pigs, as the minigenome assay did not reveal an advantage for the swine-origin internal genes.

To assess the effect of HA activity on different replication efficiencies observed in the swine cells (SD-PJEC), we evaluated the binding and entry profiles of the reassortant viruses with swapped HA in swine cells and tissues. We showed that the swine-adapted HA resulted in significantly more diffuse binding to the swine respiratory tract, particularly the lower respiratory tract, when compared to the human A/VIC/11 HA. Additionally, viruses that contained a swine-adapted HA showed a higher number of infected swine cells (SD-PJEC) within 1 h post infection, which may be correlated with the improved binding observed here. Similarly, previous work has shown that two human-origin, triple reassortant viruses at different stages of adaptation to pigs had different binding profiles, despite showing similar preferences to the SA linkage and type [29]. Swine and human IAVs typically show a binding preference to α2,6-SA [14,30] and the distribution of SA glycans in the swine respiratory tract resembles that of humans, with α2,6-SA more abundant in the upper respiratory tract [18,19]. Hence, the binding differences observed here are likely related to other differences in the glycan profiles between humans and pigs. *N*-acetylneuraminic acid (NeuAc) SA and *N*-glycolylneuraminic acid (NeuGc) SA are both expressed in the swine respiratory tract, while humans only express NeuAc due to a deletion in the cytidine mono-phospho-N-acetylneuraminic acid hydroxylase (CMAH) gene required to convert NeuAc into NeuGc [31]. It is possible that the HA mutations acquired during adaptation to swine affected the sw/MO/14 preference to different types of SA, resulting in more diffuse binding. Further studies are needed to understand the impact these specific mutations would have on binding differences between human and swine IAVs with similar SA-linkage/type preferences.

The host’s basal temperature at the primary replication site was shown to play a role in IAV replication and host restrictions. While the temperature of the intestinal tract of most avian species is around 40 °C, the temperature of the human upper respiratory tract is 33 °C to 34 °C. Consequently, human-adapted IAVs show increased replication efficiency at lower temperatures than avian-adapted viruses [25,32,33]. This difference is attributed to the optimal temperature for polymerase activity, which has been shown to be correlated with amino acid 627 in the PB2 gene [25]. The temperature of the upper respiratory tract of pigs is approximately 37 °C and 38–40 °C depending on age in the lower respiratory tract. We showed that human seasonal internal genes displayed increased replication efficiency at 33 °C both in MDCK and SD-PJEC cells, but decreased replication at 40 °C. In contrast, the internal genes of sw/MO/14 showed better replication at 37 °C and 40 °C than at 33 °C. These genes are of the 2009 H1N1 pandemic (H1N1pdm09) lineage, which was previously shown to efficiently replicate in both the upper and the lower respiratory tracts of pigs [34]. However, viruses with the human seasonal HA replicated poorly in the swine cell line regardless of the temperature tested, likely as a result of the lower attachment and entry observed here. Surprisingly, the A/VIC/11 polymerase complex showed higher activity at all temperatures compared to both swine-adapted polymerase complexes (sw/MO/14 and ty/OH/04), including at 40 °C. Although these results contradict our findings for the growth kinetics at different temperatures, it is important to highlight that our minigenome assay was performed using MDCK-SIAT1 cells, but the highest differences for growth kinetics at 33 °C or 40 °C were observed in the swine SD-PJEC cells. Nevertheless, these findings support the conclusion that other host factors as well as the combination of genes, in this case the combination of a host-adapted HA with an efficient vRNP, may be involved in increased replication of the swine-adapted virus in swine cells rather than inherent polymerase activity alone.

We found that human and swine-adapted viruses have striking differences in susceptibility to the pSP-D, a collectin of the innate immune system that has strong antiviral activity against IAVs. SP-D binds to glycans present on the HA, blocking IAV attachment to epithelial cells, resulting in non-specific virus neutralization and clearance [35]. Susceptibility to SP-D activity is associated with the glycosylation pattern of a virus: viruses containing low levels of glycosylations are more resistant to SP-D neutralization, while highly glycosylated viruses are more susceptible [36,37,38,39,40]. Here, we showed that the human seasonal virus (A/VIC/11) showed significantly greater susceptibility than the sw/MO/14 swine-adapted virus, likely a result of the increased number of putative glycosylation sites present on the human H3 HA (9 putative glycosylation sites for A/VIC/11 HA versus 7 for sw/MO/14 HA as predicted by NetNGlyc 1.0 software [41]). pSP-D was shown to exert stronger antiviral activity against different IAV strains compared to human SP-D, mainly due to structural features in the carbohydrate recognition domain (CRD) that facilitate and increase interaction with IAV HA [24,42,43]. Hence, we speculate that the mutations acquired during adaptation in pigs, notably the loss of two N-glycosylations (N133 and N165 H3 amino acid positions) that are still conserved in these viruses even a decade later, reduced the susceptibility of the sw/MO/14 HA to the action of pSP-D, contributing to its success in the swine host.

Interspecies transmission of human viruses to pigs is fairly common; however, wholly human IAV rarely become established in swine. Influenza viruses consistently acquire specific mutations and/or gene segments through reassortment to effectively replicate and transmit in a new species [16,44,45]. This is typically the case for human viruses that spillover to pigs, in which only some of the human viral gene segments persist but with mutations that differ from the precursor human strain [8,9]. Our results suggest that the combination of swine-adapted internal genes with mutations in the HA gene was essential for the adaptation of this H3N1 human-origin virus to pigs. However, further studies are needed to understand which of the individual mutations and reassorted genes were the critical changes that allowed cross-species infection and subsequent sustained transmission in pigs.

## 4. Materials and Methods

Viruses and cells: Eight viruses (Table 1) were generated by reverse genetics (rg) using an 8-plasmid system as previously described [46,47] and previously tested for pathogenesis and transmission in vivo [8]. Briefly, plasmids were generated in the bidirectional plasmid vector pDP2002 for all genes of two parental viruses, a human-origin swine H3N1 virus of the 2010.1 lineage (A/swine/Missouri/A01410819/2014; sw/MO/14) detected through the USDA IAV in swine surveillance system and a seasonal human H3N2 virus (A/Victoria/361/2011; A/VIC/11) with similar HA ancestry. Reassortant viruses were generated by swapping the surface HA and/or NA genes from either of the parental viruses, maintaining the full constellation of their internal genes- the PB2, PB1, PA, NP, M and NS segments (Table 1). Reassortants and the parental viruses were rescued in co-cultures of human embryonic kidney 293T cells and Madin-Darby canine kidney (MDCK) cells and the genetic sequences were confirmed by Sanger sequencing. A/turkey/Ohio/313053/2004 (ty/OH/04; H3N2), a swine-origin virus detected in turkeys that was shown to efficiently replicate in pigs [48], was used as a control virus for binding and polymerase assays. Virus stocks were propagated in MDCK cells. SIAT1 Stable Expressing MDCK (MDCK-SIAT1) cells were used for polymerase activity assays. South Dakota porcine jejunum epithelial cell line (SD-PJEC) was cultured following previously described conditions [49].

Replication kinetics in swine cells and trachea explants: MDCK and SD-PJEC cells were infected with reassortant and parental rg viruses at a multiplicity of infection (MOI) of 0.01 in 24-well plates at 90% confluency. Following a 1h incubation, cells were washed three times with phosphate-buffered saline (PBS) and Opti-MEM I (Life Technologies, Carlsbad, CA, USA) medium added containing 1% antibiotic-antimycotic (Sigma-Aldrich, St. Louis, MO, USA) and L-1-Tosylamide-2-phenylethyl chloromethyl ketone (TPCK)-treated trypsin (1 μg/mL for MDCK and 0.1 μg/mL for SD-PJEC). Cells were incubated at 33 °C, 37 °C or 40 °C in 5% CO_2_ and cell culture supernatants were collected at 12, 24, 48, and 72 h post incubation (hpi), and virus titers were determined by 50% tissue culture infective dose (TCID_50_) as previously described [50].

Tracheas were obtained from 5-week-old crossbred pigs from a healthy herd confirmed to be seronegative to IAV antibodies. Pigs were humanely euthanized with a lethal dose of pentobarbital (Fatal Plus; Vortech Pharmaceuticals, Dearborn, MI, USA). The whole tracheobronchial structure was collected and prepared as previously described with modifications [51,52]. Briefly, the tracheas were washed three times in a warm 1:1 mixture of Dulbecco’s modified Eagle’s medium (DMEM, Sigma-Aldrich, St. Louis, MO, USA) and RPMI 1640 medium (Sigma-Aldrich, St. Louis, MO, USA) supplemented with 1% antibiotic-antimycotic (Sigma-Aldrich, St. Louis, MO, USA), divided in two lengthwise, and sections cut with disposable 8-mm biopsy punches. Tissue punches were placed with the epithelium facing up on fine-meshed gauze in 24-well plates and cultured at 37 °C and 5% CO_2_ with 600 µL of 50% RPMI/50% DMEM mixture with 1% antibiotic-antimycotic so they were slightly submerged. Explants were transferred to 48-well plates (on fine-meshed gauze) after 18h incubation and inoculated with 10^6^ TCID_50_ of each virus in a volume of 300 μL. After 1h incubation at 37 °C, explants were washed three times with warm PBS and 300 μL of RPMI/DMEM mixture with antibiotic-antimycotic was added. Supernatants were collected at 12, 24, 48, and 72 hpi.

Virus histochemistry in swine respiratory tissue: Nasal turbinate, tonsil crypts, trachea, and lung tissues from 3 influenza virus- and antibody-negative, naïve 5-week-old pigs were used for virus histochemistry as described previously [53]. Concentrated sw/MO/14rg, A/VIC/11rg, and a swine-origin control virus (ty/OH/04) were inactivated by 1% formalin and labeled with fluorescein isothiocyanate (FITC) by mixing equal volumes of each virus and freshly prepared 0.1 mg/mL FITC in 0.5 M bicarbonate buffer (pH 9.5), followed by dialysis in PBS. Deparaffinized and rehydrated formalin-fixed and paraffin-embedded (FFPE) respiratory tissues were incubated with the FITC-labeled influenza viruses (100 hemagglutination units [HAU]/50 μL) overnight at 4 °C in a humidified chamber. Virus-attachment was then detected after washing with 0.2 M Tris-HCl, 0.1 M NaCl, 0.5% Tween 20 (TNT) buffer using peroxidase-labeled anti-FITC antibody (Dako, Glostrup, Denmark) and the tyramide signal amplification (TSA) biotin system following manufacturer’s recommendation (PerkinElmer, Waltham, MA, USA). Slides were stained with 3-Amino-9-ethyl-carbazole (AEC; Sigma-Aldrich, St. Louis, MO, USA) and counterstained with Mayer’s hematoxylin. The distribution of viral binding to the surface epithelium of the respiratory tract was scored from 0 (no binding) to 4 (intense, diffuse binding) by a pathologist blinded to experimental treatments.

Hemagglutination Inhibition (HAI) Assay: Synthetic recombinant porcine surfactant protein-D (pSP-D; Genbank accession- NP_999275), a collectin involved in innate immune defense, was expressed by Life Technologies (Life Technologies, Carlsbad, CA, USA) in FreeStyle™ HEK293 mammalian cells as a custom service. An HAI assay was used to compare the susceptibility of the different viruses to pSP-D as previously described [54], with some modifications. The synthetic pSP-D was two-fold serially diluted in PBS containing 1 mM of CaCl_2_ and 0.5 mM of MgCl_2_ (PBS++; 25 µL) in round-bottom 96-well plates. pSP-D concentrations ranged from 215 µg/mL to 0.21 µg/mL. Each concentration of pSP-D was incubated with 4 HAU of sw/MO/14rg or A/VIC/11rg in equal volumes and incubated for 30 min at room temperature. Then, 50 µL of 0.5% turkey red blood cells suspension in PBS++ were added and plates were incubated for 2 h at room temperature. Results are expressed as the minimal concentration of pSP-D required to fully inhibit agglutination. The HAI was performed in triplicate in two independent experiments.

Indirect Immunofluorescence: Indirect immunofluorescence analysis was carried out with MDCK cells and SD-PJEC in 96-well plates. Cells at 90% confluency were infected in triplicate at an MOI of 10 in triplicates with sw/MO/14rg, VIC11-HA, A/VIC/11rg, or MO14-HA in Opti-MEM with TPCK and incubated at 37 °C in 5% CO_2_. At 1 h post-infection (hpi), supernatants were removed and cell monolayers were fixed with 4% paraformaldehyde in PBS for 15 min and permeabilized with 0.5% Triton X-100 (Sigma-Aldrich, St. Louis, MO, USA) in PBS for 5 min. Cells were incubated for 1 h with swine polyclonal serum (1:1000) against either sw/MO/14 or A/VIC/11 produced in previous studies [8], followed by 1 h incubation with the secondary FITC-labeled anti-swine IgG antibody (1:300; Southern Biotech, Birmingham, AL, USA). Images were acquired with a Leica DM IRBE microscope with a Leica DFC500 digital camera and the Leica Application Suite version 3.7.0 (Leica Microsystems, Wetzlar, Germany) at magnifications of 200× and 400×.

Flow Cytometry: To quantify the attachment and entry of the reassortant viruses into the cells, MDCK cells and SD-PJEC in 12 well plates at 90% confluency were infected at an MOI of 5 in duplicates with each of the 8 rg viruses in Opti-MEM with 1 μg/mL TPCK. After 1 h incubation at 37 °C in 5% CO_2_, cells were harvested by gently scraping with a rubber tipped cell scraper (BD Biosciences, Franklin Lakes, NJ, USA) and fixed and permeabilized using BD Cytofix/Cytoperm (BD Biosciences, Franklin Lakes, NJ, USA) following the manufacturer’s protocol. To quantify virus entry, cells were incubated for 15 min with a mouse anti-Influenza A virus monoclonal antibody (1:150; HB65, ATCC, Manassas, VA, USA) followed by R-phycoerythrin (PE) conjugated anti-mouse IgG2a antibody (5 μg/mL; Southern Biotech, Birmingham, AL) for 15 min. Data were acquired using an LSRII with FACSDiva software (BD Biosciences, Franklin Lakes, NJ, USA) and analysis was performed with FlowJo 4.6 software (Treestar, Ashland, OR, USA). At least 10,000 events were analyzed for each sample.

Polymerase activity assay: A minigenome reconstitution assay was used to assess polymerase activity using the pHW-SP reporter plasmid containing the Gaussia luciferase (GLuc) open reading frame flanked by the non-coding regions of NS segment from A/guinea fowl/Hong Kong/WF10/1999. The pEGFP-C1 vector was used for the different PB1, PB2, PA, or NP segments after removal of the EGFP. The minigenome assays were performed as previously described [55], with modifications. Briefly, MDCK-SIAT1 cells at 70–90% confluency in 24-well plates were transfected with 1 μg of the reporter plasmid, in addition to 1 μg each of the plasmids encoding the polymerase complex (PB1, PB2, PA, and NP) from sw/MO/14, A/VIC/11, or ty/OH/04. The pCMV-SEAP plasmid, which contains a secreted alkaline phosphatase (SEAP) gene under the control of the of the cytomegalovirus promoter (pCMV), was co-transfected into the cells to normalize transfection efficiency as described previously [56]. Transfections were performed using 2:1 Lipofectamine 2000 transfection reagent to DNA (ThermoFisher Scientific, Waltham, MA, USA) according to the manufacturer’s recommendations. Cells were incubated at 33°, 37° or 40 °C in 5% CO_2_ and at 12, 24, 48, and 72 h post-transfection, the supernatants were collected and stored at −80·°C until assayed. Luciferase activity was measured using the Pierce Gaussia Luciferase Glow Assay (ThermoFisher Scientific, Waltham, MA, USA) according to the manufacturer’s protocol. Secreted alkaline phosphatase activity was measured at 12h post-transfection by the Phospha-Light SEAP reporter gene assay system (Life Technologies, Carlsbad, CA, USA). GLuc and SEAP signals were measured using an EnSight multimode plate reader (PerkinElmer, Waltham, MA, USA). Background signal was removed by measuring signal from non-transfected wells. Relative polymerase activity was calculated as a ratio of GLuc activity minus cell background divided by SEAP activity multiplied by 1000. Experiments were performed twice using triplicate wells.

Statistical analysis: All statistical analyses were conducted by using GraphPad Prism version 9.3.1 (GraphPad Software, San Diego, CA, USA). A p-value below 0.05 was considered significant. Growth kinetics and polymerase assay data were compared using a two-way analysis of variance (ANOVA) for multiple comparisons with post hoc Tukey test. Entry flow cytometry and SP-D hemagglutination inhibition data were compared using two-tailed Student’s *t* test.

## Figures and Tables

**Table 1:** Genetic composition of reassortant viruses generated using the human seasonal H3N2 influenza A virus A/Victoria/361/2011 (A/VIC/11), and the H3N1 human-origin A/swine/Missouri/A01410819/2014 (sw/MO/14).

Virus	HA Gene Origin	NA Gene Origin	Internal Genes Origin
sw/MO/14rg	sw/MO/14	sw/MO/14	sw/MO/14
VIC11-HA	A/VIC/11	sw/MO/14	sw/MO/14
VIC11-NA	sw/MO/14	A/VIC/11	sw/MO/14
VIC11-HA/NA	A/VIC/11	A/VIC/11	sw/MO/14
A/VIC/11rg	A/VIC/11	A/VIC/11	A/VIC/11
MO14-HA	sw/MO/14	A/VIC/11	A/VIC/11
MO14-NA	A/VIC/11	sw/MO/14	A/VIC/11
MO14-HA/NA	sw/MO/14	sw/MO/14	A/VIC/11

HA = hemagglutinin; NA = neuraminidase.

## Data Availability

Not applicable.
